# Jaw Fibro-Osseous Lesions: Use of a Predictive Index in Grading Probable Malignant Changes and a Review of Cases

**DOI:** 10.30476/dentjods.2023.96389.1935

**Published:** 2024-03-01

**Authors:** Akindayo Olufunto Akinyamoju, Seyi John Akinloye, Robinson Obos Okiti, Bukola Folasade Adeyemi

**Affiliations:** 1 Dept. of Oral Pathology, University of Ibadan, University College Hospital, Ibadan, Oyo State, Nigeria; 2 Dept. of Oral Pathology, University College Hospital, Ibadan, Oyo State, Nigeria

**Keywords:** Fibro-osseous neoplasms, Osteogenic Sarcoma, Predictive, Grading, Malignancy

## Abstract

**Statement of the Problem:**

Fibro-osseous lesions (FLs), may rarely exhibit malignant features likewise undergo malignant transformation. Awareness of these features can assist in screening for potentially malignant cases and identifying low-grade central osteogenic sarcoma (LGCOS) that may mimic FLs.

**Purpose:**

The objective of this study was to determine the usability of an index in predicting malignant changes in jaw FLs

**Materials and Method:**

This was a retrospective study where hematoxylin and eosin (H&E) slides and archival records of fibrous dysplasia (FD) and ossifying fibroma (OF) cases were reviewed. The sections were assessed for permeation of marrow spaces, stromal growth pattern, cytologic atypia, mitotic activity, and pattern of bone growth, which are parameters for diagnosing LGCOS. The predictive histologic index of malignancy (PHIM) was determined by a sum of the scores and graded as 0=nil, 1=low, 2 & 3=moderate, and 4 & 5=high. Data were presented using descriptive analysis.

**Results:**

Ninety-three cases of FLs met the inclusion criteria, consisting of 40(43%) cases of FD and 53(57%) cases of OF. The peak age of presentation for FD and OF was 2^nd^ and 3^rd^ decade. There was a female preponderance of 1:1.6. The maxilla was the most common site affected by FD, while the mandible was most commonly affected by OF. For FD cases, the PHIM was moderate in 10(25%) cases and low in 21(52.5%) cases. Similarly, for OF cases, 30(56.6%) cases had low grade PHIM while 10(17%) cases had moderate grade PHIM.

**Conclusion:**

The PHIM depicted low to moderate malignancy grade in some of the cases studied. Follow up studies would be necessary to assess the PHIM.

## Introduction

Fibro-osseous lesions (FLs) are a group of benign lesions characterized by replacement of bone by fibrous tissue, which later undergoes varying degrees of calcification [ 1 ]. Common amongst FLs are fibrous dysplasia (FD) and ossifying fibroma (OF) [ 1 - 3 ]. It is believed that FD is due to postzygotic somatic activating missense mutations in the GNAS 1 (guanine nucleotide binding protein alpha stimulating activity polypeptide 1) gene [ 4 - 7 ]. FD is a diffuse slow growing neoplasm of bone [ 1 - 3 ]; the monostotic type has no gender predilection while polyostotic FD has a female gender predilection [ 8 - 9 ]. It commonly affects the jaws with preference for the maxilla, as well as the skull, ribs, and femur [ 10 ]. Histology typically shows foci of irregularly shaped trabeculae of immature (woven) bone in a moderately cellular fibrous connective tissue stroma. The bony trabeculae are curvo-linear shaped, giving them the Chinese lettering appearance. Osteoblastic rimming is absent or minimal, and peri-trabecular clefting is common [ 11 - 12 ]. Lamellar bone may be seen in mature lesions and mitotic figures are absent [ 10 - 12 ]. 

Similarly, OF is a slow growing painless expansile neoplasm of bone [ 13 ] showing predilection for females [ 1 - 3 ]. Most lesions are located in the posterior region of the mandible [ 14 ]. Histologically, OF is mainly composed of a fibrous stroma interspersed by woven or lamellar bony trabeculae as well as cementum-like spherules, demonstrating osteoblastic rimming and varying degrees of maturation [ 13 ].

Though FLs are benign in nature with a low risk of malignant transformation, they have been reported to co-exist with, or transform to low grade central osteogenic sarcoma (LGCOS) [ 15 - 17 ]. This is a rare subtype of osteosarcoma that is less aggressive than the conventional osteosarcoma [ 15 - 17 ]. It can mimic FLs (in particular FD) radiographically and microscopically [ 16 - 17 ]. The histology of LGCOS shows stromal proliferation with spindling and production of irregular bone. There is minimal cellularity, mild cellular atypia, and few mitotic figures, features that may be seen in FLs. Also, when LGCOS is well differentiated, it could be mistaken for FLs [ 16 - 17 ]. Likewise, the bone distribution pattern of LGCOS could mimic that of FD [ 16 ]. 

Though malignant transformation of FLs may occur asymptomatically, some clinical features may mark this transformation: rapid painful growth, neurological disturbances, cortical destruction, and so on. [ 18 ]. However, it is desirable that the propensity for malignant change be predictable and quantifiable on histological assessment of biopsy and surgical samples. Awareness of the histopathological features of malignancies seen in a transformed tumor, for instance, LGCOS, can assist in identifying and grading FLs with potential for malignant change. The use of a grading index using the morphological features of malignancy seen in LGCOS could be applied to screening for malignancy in FLs. This would ensure early identification and proper management of such cases. Thus, this study sets out to determine the usability of an index in predicting malignant changes in jaw fibro-osseous lesions.

## Materials and Method

This retrospective study was performed at the Oral Pathology Department at our institution. The hematoxylin and eosin (H&E) slides and archival records of FLs, consisting of FD and OF cases, diagnosed for the period 2005 to 2019, were retrieved and reviewed. The inclusion criteria were adequacy of records and availability of their formalin fixed paraffin embedded (FFPE) tissue. Cases with incomplete records or inadequate tissue on the wax blocks were excluded from further consideration in the study. Furthermore, new H&E sections were made from the wax blocks and these were assessed by one of the authors (AAO) to verify the diagnosis of either FD or OF. Also, two of the authors (ORO and ASJ) independently assessed the slides for the following morphological characteristics described by Inward [ 16 ] in recognizing malignancy in FD in order to differentiate FD from LGCOS. The features included permeation of marrow spaces, stromal growth pattern (intersecting bundles of spindling fascicles), cytologic atypia, mitotic activity, and pattern of bone growth (large bony growth pattern). These were considered to be the morphologic features that would constitute the index. A score of 1 was given where any of these morphologic features were present while a score of 0 was recorded when absent. In cases where the opinion of the two authors differed, the disagreement was resolved after a joint review, and a consensus on each case was reached with a third reviewer (ABF). The index, subsequently referred to as the Predictive Histologic Index of Malignancy (PHIM) was determined by a sum of the scores and graded as follows. According to their risk for malignancy a total score of 0 was assumed to have no PHIM (no risk); a total score of 1 was assigned a low PHIM grade (low risk); a total score of either 2 or 3 was assigned a moderate PHIM grade (moderate risk), and total scores of either 4 or 5 were assigned a high PHIM grade (high risk). Data were presented using descriptive analysis. Ethical approval for this study was obtained from the Ethical Review Board of our institution (UI/EC/21/0083).

## Results

Ninety-three cases of FLs met the inclusion criteria consisting of 40(43%) cases of FD and 53(57%) cases of OF. The mean ages for FD and OF were 26.2±12.8 years and 33.1±14.1 years respectively. The peak age of presentation for FD was in the 2^nd^ decade with 15(37.5%) cases while OF recorded a peak age of presentation in the 3^rd^ decade with 14(26.4) cases.

The M: F ratio recorded for both FD and OF was 1: 1.6, inferring female gender preponderance. In addition, the maxilla was the most common site affected by FD, recording 26(65%) cases while the mandible was most commonly affected by OF, representing 33(62.3) cases (Table 1).

**Table 1:** Distribution of PHIM grade in FLs by age group, gender and site of tumor

	Predictive grade of tumor			Total n (%)	*p* Value
	No n (%)	Low n (%)	Moderate n (%)		
Age group (years)					
Fibrous dysplasia					
0-28	5(55.6)	11(52.4)	9(90.0)	25 (62.5)	0.12
>28	4(44.4)	10(47.6)	1(10.0)	15(37.5)	
Ossifying fibroma					
0-28	5(35.7)	13(43.3)	4(44.4)	22 (41.5)	0.93
>28	9(64.3)	17(56.7)	5(55.6)	31 (58.5)	
Gender					
Fibrous dysplasia					
Male	4(44.4)	6(28.6)	5(50.0)	15 (37.5)	0.46
Female	5(55.6)	15(71.4)	5(50.0)	25 (62.5)	
Ossifying fibroma					
Male	3(21.4)	11(36.7)	6(66.7)	20 (37.7)	0.09
Female	11(78.6)	19(63.3)	3(33.3)	33 (62.3)	
Site of tumor					
Fibrous dysplasia					
Maxilla	4(44.4)	14(66.7)	8(80.0)	26 (65.0)	0.25
Mandible	4(44.4)	6(28.6)	1(10.0)	11 (27.5)	
Maxillo-mandible	1(11.1)	1(4.9)	-	2 (5.0)	
Cranio-facial	-	-	1(10.0)	1 (2.5)	
Ossifying fibroma					
Maxilla	6(42.9)	13(43.3)	1(11.1)	20 (37.7)	0.21
Mandible	8(57.1)	17(56.7)	8(88.9)	33 (62.3)	
Maxillo-mandible	-	-	-		
Cranio-facial	-	-	-		

*p* Values based on chi square test (Fisher’s exact)

**Figure 1:**
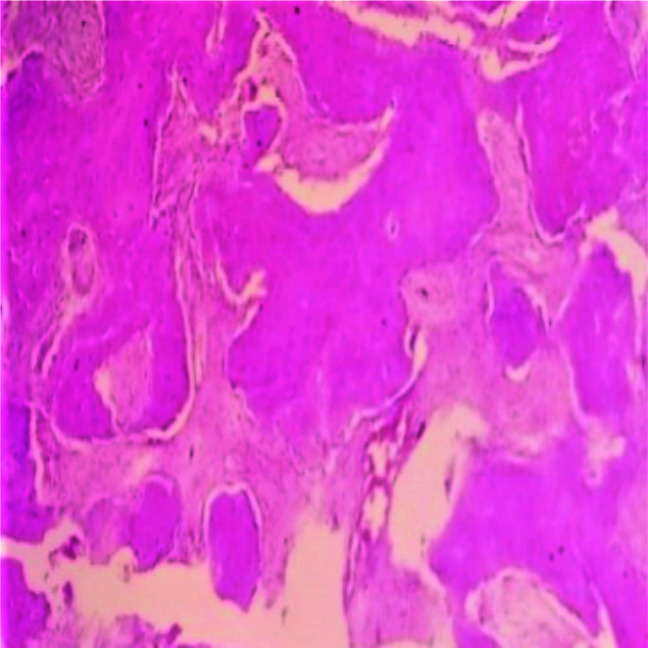
Section shows large bone trabeculae growth pattern H & E 40×

**Figure 2:**
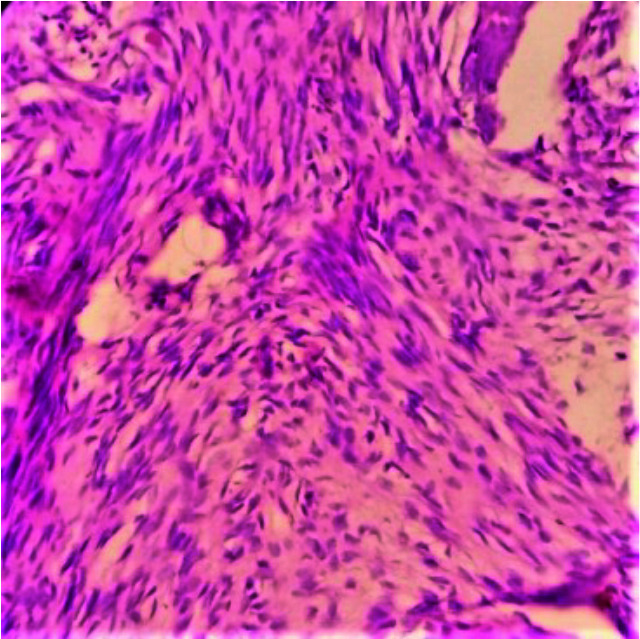
Section shows intersecting bundles of spindling fascicles H & E 100×

**Figure 3:**
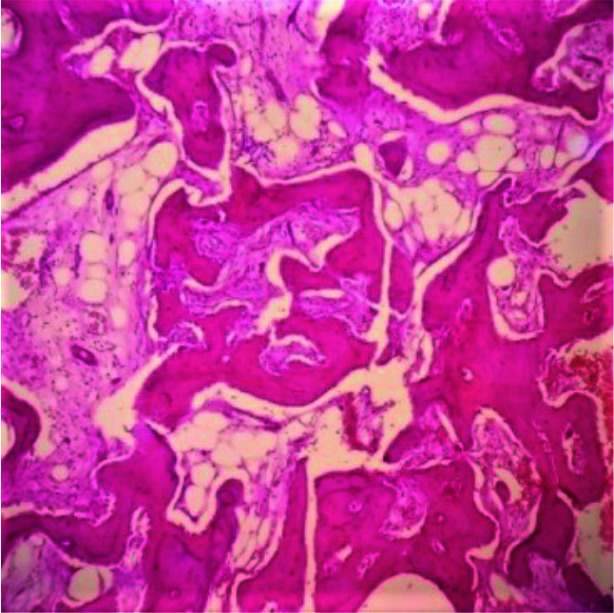
Section shows permeation of marrow spaces by bony trabeculae H & E 40×

**Figure 4:**
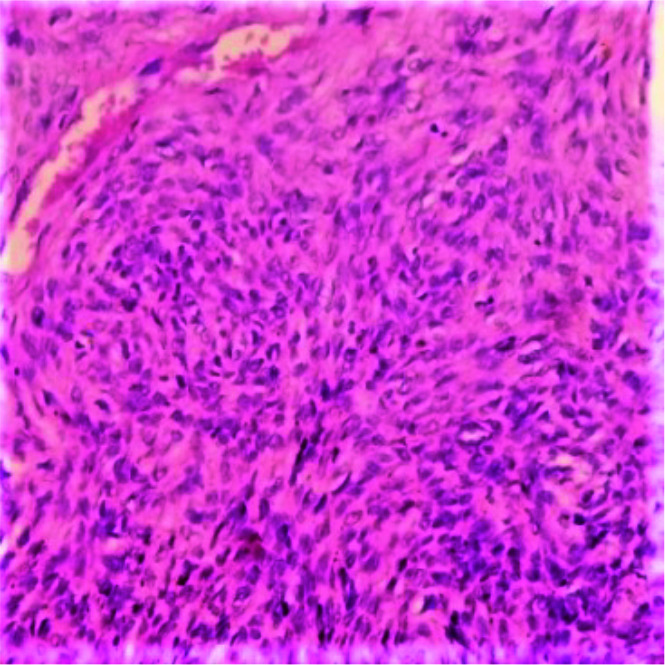
Section shows mild cellular atypia with vesicular nuclei H & E 100×

Furthermore, the distribution of the predictive features of malignancy in FD cases were as follows: presence of large bone growth pattern– 20(50%) cases (Figure 1); presence of intersecting bundles of spindling fascicles -16(40%) cases (Figure 2); presence of permeative growth pattern -3(7.5%) cases (Figure 3) and presence of mild cytologic atypia-3(7.5%) cases (Figure 4). Also, for the OF cases; large bony growth patterns were seen in 9(17%) cases, intersecting bundles of spindling fascicles were seen in 34(64.2%) cases and mild cellular atypia in 6(11.3%) cases. No features of permeative growth pattern were seen in OF cases while mitotic figures were not seen in both FD and OF cases. 

Following the analysis of the sum of the individual scores obtained for FD cases, 9(22.5%) cases had no morphologic feature mimicking a malignant pattern, thus obtaining a total score of 0 and designated as no PHIM grade (no risk). However, 21(52.5%) cases had a total score of 1 and were designated as low-grade PHIM (low risk); 9(22.5%) cases and 1(2.5%) case obtained a total score of 2 and 3 respectively and were designated as moderate grade PHIM (moderate risk). Similarly, for OF cases, 14(26.4%) cases had no morphologic feature of a malignant pattern, thus obtaining a total score of 0 and designated as no PHIM grade (no risk). Also, 30(56.6%) cases which had a total score of 1 were designated as low-grade PHIM (low risk) while 8(15.1%) cases and 1(1.9%) case with total scores of 2 and 3 respectively were designated as moderate grade PHIM (moderate risk) (Table 1). No case had a total score of 4 and above, thus none had a high-grade PHIM (high risk). There was no statistically significant difference in the PHIM for FD and OF cases (*p*= 0.63), as well as for age group (*p*= 0.12 and *p*= 0.93), gender (*p*=0.46 and 0.09) or site (*p*= 0.25 and 0.21) of the respective FD and OF cases (Table 1).

## Discussion

The scoring of tissue sections can be a beneficial way of patients’ assessment for treatment purposes, research, and substantiating morphologic conclusions [ 19 ]. In particular, suspicious FLs lesions can be flagged early, and treatment instituted promptly. However, comparison of this study with previous studies was challenging due to dearth of studies exploring the use of an index in determining malignant change in FLs. Nevertheless, some similarities and a few differences were seen in the clinico-demographic features of FLs in the present study and previous studies. In this study, OF cases were the more prevalent FL, which was in agreement with previous studies [ 20 - 21 ]. The mean age for FD in this study was 26.2±12.8 years, which was similar to findings by Dube *et al*. [ 20 ]. Also, mean age of 33.1±14.1 years for OF in this study, was similar to what was obtained in previous studies [ 22 - 23 ]. This finding was however different from the mean age for OF obtained from other studies which recorded a younger mean age [ 20 , 21 ]. The peak age of presentation for FD in this study was in the 2^nd^ decade. This slightly differed from findings in the study by Moshy *et al*., who recorded 2^nd^ and 3^rd^ decade [ 21 ]. In addition, this study recorded a peak age of 3^rd^ decade for OF which slightly differed from findings in previous studies [ 20 - 21 , 24 ].

Generally, in this study, females were the predominant gender affected by FLs, though the proportion was higher for OF. This was in agreement with previous studies, corroborating known literature on female preponderance of OF [ 20 - 24 ]. Similarly, this study recorded a female preponderance in FD, which was in keeping with a previous study by Davidora *et al*. [ 25 ]. In addition, the site distribution of FLs in the present study was in keeping with findings from previous studies; the maxilla was the more commonly affected jaw in FD [ 20 - 21 , 25 ]. Moreover, the mandible was the more common site for OF, which agreed with previous reports [ 21 - 24 ]. However, this finding differed from that of Dube *et al*. [ 20 ], who reported a preference for the maxilla for OF cases. The minor differences in comparing the clinico-demographic features of this study with those of previous studies may be due to the population sampled. Nonetheless, the features seen in this study are largely in line with known features of FLs.

Typically, FLs are benign and are not expected to have dysplastic or malignant features. However, subtle features, reminiscent of patterns seen in LGCOS or low-grade fibrosarcoma may be seen in FLs. [ 16 ]. This study showed some of these features were present in varying amounts in the FLs studied, though were low in prevalence. Also, in this study, the most prevalent predictive factors were intersecting bundles of spindling fascicles for OF and large bone growth pattern for FD. Other features like marrow permeation and mild cellular atypia were also seen. These features may be helpful in reaching a correct diagnosis or alerting the surgeon to a more intense patient follow-up. This is because spindling fascicles and marrow permeation are not typical features of FLs. Thus, when these features are prevalent, the probability of the tumor being malignant or undergoing malignant transformation may be higher.

Although previous case reports have reported both FD and OF to have transformed to a malignancy [ 17 , 26 - 27 ], it was challenging in this study to ascertain which of FD or OF was more likely to transform. In spite of FD having a higher proportion of permeative and large bone growth pattern, OF exhibited a higher proportion of intersecting bundles of spindling fascicles and cellular atypia. Also, histopathologic features of malignancy described in a previous case report of benign FLs which transformed, include high cellularity and infiltrative growth pattern [ 17 ]. However, FD has been reported to have a substantial risk of malignant change, in a systematic review of FLs with malignant transformation [ 27 ]. 

Furthermore, several advanced techniques have been reported to be useful in determining malignant change in FLs. This includes a positive GNAS mutation status in a FD derived OS [ 28 - 30 ]. In addition, immunohistochemical analysis of murine double-minute type 2 and cyclin-dependent kinase 4 (CDK4) can help to distinguish low-grade osteosarcoma from benign histological imitators [ 31 - 32 ], since FLs do not express them. Similarly, oncogenes associated with osteosarcoma: c-myc, c-fos, and c-jun, are not expressed in FLs [ 33 ]. These are often expensive and not routinely used in resource-limited settings. Histopathologic assessment remains an important hallmark of diagnosis in our hospital and most hospitals in our country. Thus, the identification of histological features that could distinguish FLs from LGCOS would be of immense benefit in patient management. 

Subsequently, this study assessed previously diagnosed FLs for features of malignancies using the PHIM grading system. Though the cases available over the study period may constitute a majority of FLs, applicability of the PHIM was constrained by loss of patients to follow up and unavailability of records identifying FLs that could have subsequently developed malignant tendencies, clinically and histologically. This would have aided in evaluating the applicability of this grading system.

## Conclusion

In this study, the PHIM depicted low to moderate malignancy risk in some of the cases studied. This finding should however be approached with caution because this is a preliminary study and first of its type. More studies with larger sample sizes of suspicious cases and known transformed cases, as well as follow up studies would be necessary to assess the value of the PHIM. 
